# The NIEHS Superfund Research Program: 25 Years of Translational Research for Public Health

**DOI:** 10.1289/ehp.1409247

**Published:** 2015-05-15

**Authors:** Philip J. Landrigan, Robert O. Wright, Jose F. Cordero, David L. Eaton, Bernard D. Goldstein, Bernhard Hennig, Raina M. Maier, David M. Ozonoff, Martyn T. Smith, Robert H. Tukey

**Affiliations:** 1 Icahn School of Medicine at Mount Sinai, New York, New York, USA; 2 University of Puerto Rico Graduate School of Public Health, San Juan, Puerto Rico; 3 School of Public Health, University of Washington, Seattle, Washington, USA; 4 Graduate School of Public Health, University of Pittsburgh, Pittsburgh, Pennsylvania, USA; 5 University of Kentucky College of Agriculture, Food and Environment, Lexington, Kentucky, USA; 6 Department of Soil, Water and Environmental Science, University of Arizona, Tucson, Arizona, USA; 7 Boston University School of Public Health, Boston, Massachusetts, USA; 8 School of Public Health, University of California, Berkeley, Berkeley, California, USA; 9 University of California, San Diego, La Jolla, California, USA

## Abstract

**Background:**

The Superfund Research Program (SRP) is an academically based, multidisciplinary, translational research program that for 25 years has sought scientific solutions to health and environmental problems associated with hazardous waste sites. SRP is coordinated by the National Institute of Environmental Health Sciences (NIEHS). It supports multi-project grants, undergraduate and postdoctoral training programs, individual research grants, and Small Business Innovation Research (SBIR) and Technology Transfer Research (STTR) grants.

**Results:**

SRP has had many successes: discovery of arsenic’s toxicity to the developing human central nervous system; documentation of benzene toxicity to hematologic progenitor cells in human bone marrow; development of novel analytic techniques such as the luciferase expression assay and laser fragmentation fluorescence spectroscopy; demonstration that PCBs can cause developmental neurotoxicity at low levels and alter the genomic characteristics of sentinel animals; elucidation of the neurodevelopmental toxicity of organophosphate insecticides; documentation of links between antimicrobial agents and alterations in hormone response; discovery of biological mechanisms through which environmental chemicals may contribute to obesity, atherosclerosis, diabetes, and cancer; tracking the health and environmental effects of the attacks on the World Trade Center and Hurricane Katrina; and development of novel biological and engineering techniques to facilitate more efficient and lower-cost remediation of hazardous waste sites.

**Conclusion:**

SRP must continue to address the legacy of hazardous waste in the United States, respond to new issues caused by rapid advances in technology, and train the next generation of leaders in environmental health science while recognizing that most of the world’s worst toxic hot spots are now located in low- and middle-income countries.

**Citation:**

Landrigan PJ, Wright RO, Cordero JF, Eaton DL, Goldstein BD, Hennig B, Maier RM, Ozonoff DM, Smith MT, Tukey RH. 2015. The NIEHS Superfund Research Program: 25 years of translational research for public health. Environ Health Perspect 123:909–918; http://dx.doi.org/10.1289/ehp.1409247

## Introduction

Twenty-five years ago, the U.S. Congress first set aside funds to address fundamental research needs for the nation’s hazardous waste problem with the Comprehensive Environmental Response, Compensation, and Liability Act (CERCLA) of 1980 [U.S. Environmental Protection Agency (EPA) 2011]. At the time, the United States was galvanized by the discovery of a massive chemical waste disposal site at the Love Canal in Niagara Falls, New York (New York State Department of Health 1981), an event that forcefully put the legacy of many years of improper waste disposal on the public agenda. The Love Canal, an unused conduit between Lake Erie and Lake Ontario, had been used by the Hooker Chemical Company (later a subsidiary of the Occidental Petroleum Company) since the 1940s as a dumping ground for toxic waste. Once filled with chemical waste, the canal was covered with a clay seal in 1953, and homes and a school were built atop it. The waste did not stay underground. The canal filled with water, and by 1976 heavy rains regularly caused toxic sludge to bubble up into the basements of the overlying homes. Waste chemicals also contaminated nearby streams. By the time it was recognized as a hazardous waste site, Love Canal contained an estimated 21,000 tons of discarded chemicals consisting of “caustics, alkalines, fatty acids and chlorinated hydrocarbons” and was linked to a high rate of miscarriages, birth defects, and other health disorders in surrounding neighborhoods (New York State Department of Health 1981). Within a few years a second major waste site was discovered near Louisville, Kentucky. Known as the “Valley of the Drums,” the site contained thousands of 55-gal drums full of chemical wastes that had accumulated over many decades.

At that time, it became clear to policy makers and the American public that hazardous waste was an environmental and public health emergency. In response, the U.S. Congress passed CERCLA on 11 December 1980. This law became known as the Superfund Act because it authorized the creation of a large fund supported by a tax on the chemical manufacturing and petroleum industries, the two major producers of toxic wastes. Many of the new sites then being recognized were the result of actions by parties long gone. The purpose of the tax was to provide resources to remediate these orphaned sites.

One of the first actions taken under the Superfund Act was the expenditure of more than $100 million to clean up dioxin-contaminated waste oil that had been dumped to control dust on dirt roads in the rural community of Times Beach, Missouri. The U.S. EPA purchased the entire town for $35 million and then bulldozed it down. Huge incinerators were built to burn not only all of the homes and belongings in the community, but the topsoil from the dirt roads (Hernan 2010).

The Superfund tax on the oil and chemical industries expired in 1995. Today, cleanup of hazardous waste sites is funded through general tax revenues, but the other important components of CERCLA have survived. In 1986, major enhancements were put in place via the Superfund Amendments and Reauthorization Act (SARA) of 1986 (U.S. EPA 1986), the version of the law currently in force. The U.S. EPA was assigned responsibility for identifying, listing, cleaning up, and remediating hazardous waste sites through its Office of Solid Waste and Emergency Response (OSWER). The list was known as The National Priorities List (NPL) (U.S. EPA 2013b). A new agency, the Agency for Toxic Substances and Disease Registry (ATSDR), was created under the act and assigned responsibility for conducting health assessments of populations living near hazardous waste sites, with special emphasis on children. ATSDR is co-located in Atlanta, Georgia, with the Centers for Disease Control and Prevention (CDC).

*The Superfund Research Program (SRP)*. SRP was launched in 1987. It was established by the Congress to provide scientific support for the U.S. EPA and ATSDR hazardous waste programs and funded through a small set-aside from the Superfund Trust. SRP’s mission is to “seek solutions to the complex health and environmental issues associated with the nation’s hazardous waste sites with the ultimate goal of improving public health.” It has worked to provide answers to important scientific questions: How clean must a site be? And how might a site be remediated to maximum effect and at minimum cost? Chemical mixtures posed a particular challenge because hazardous waste sites often contain complex collections of chemicals, and unexpected interactions can lead to unforeseen forms of toxicity.

In this report we present a summary of the first 25 years of SRP. It is based on remarks presented at the Twenty-Fifth Annual SRP Meeting [National Institute of Environmental Health Sciences (NIEHS) 2012].

Early on, it was decided that, although funding for hazardous waste research was appropriated to the U.S. EPA, the research program would be administered by the National Institutes of Health (NIH) and conducted in the nation’s leading research universities. Accordingly, the NIEHS, a component of NIH, was assigned responsibility for establishing the program, named initially the NIEHS Superfund Hazardous Substances Basic Research and Training Program (NIEHS 1987).

In the early years of the program, research funds were awarded to EPA and then “passed through” to NIEHS. Later, to enhance administrative efficiency, this arrangement was changed to a “line item” budget allocation in the annual budget for NIEHS.

From its outset, SRP has been guided by a clear vision set by its founding director, William Suk, and nurtured and encouraged by a succession of NIEHS Directors, starting with David Rall and followed by Kenneth Olden, David Schwartz, Interim Director Sam Wilson, and current Director, Linda Birnbaum. William Suk remains the SRP Director, accounting for the stability and steadiness of the program (Wilson 2014).

In 1987, the first year of the program, four Universities were funded: University of California (UC), Berkeley; UC Davis; University of Washington; and Massachusetts Institute of Technology (MIT). Today, SRP supports research in 18 university-based Centers across the United States. These Centers are selected through rigorously independent and highly competitive peer review.

What appears at first sight to be a conventional multi-project research structure is in reality anything but conventional. One unique aspect is the requirement for an integrated suite of research projects that includes a minimum of two biomedical projects and two environmental science or engineering projects (Suk and Anderson 1999; Suk et al. 1999). There may be as many as 12 components organized around a common theme and supported by core facilities. The mandatory pairing of environmental research with biomedical research is unique in the NIH portfolio.

Each SRP must include *a*) an Administrative Core that promotes cross-disciplinary interactions, and is responsible for oversight and liaison; *b*) a Research Translation Core that disseminates information to other scientists, policy makers, and the public, helping to transform scientific discoveries into applications that can inform risk assessment and guide site remediation and disease prevention; *c*) a Community Engagement Core that builds partnerships with affected communities (Pezzoli et al. 2007); and *d*) a Training Core, essential for developing a new generation of scientists schooled in the interdisciplinary research required to solve the complex problems of abandoned hazardous wastes.

The SRP also provides funding for individual research grants (R01s) as well as for Small Business Innovation Research (SBIR)/Small Business Technology Transfer Research (STTR) grants. These Small Business grants are designed to foster the commercialization of relevant technologies and devices. The R01 program is dedicated to relevant environmental research. A parallel program at NIEHS, also funded by the Superfund appropriation, supports a national worker training curriculum and has trained 1.4 million hazardous waste workers.

The heightened vulnerability of children to environmental health threats is a theme long emphasized by SRP [National Research Council (NRC) 1993; U.S. EPA 1996]. This emphasis has resulted in extensive support for research in children’s environmental health (Landrigan et al. 1999), exemplified by the fact the NIEHS’s national network of Centers for Children’s Environmental Health and Disease Prevention Research had its origins within the UC Berkeley SRP. Several NIEHS Children’s Environmental Health programs are now co-located in universities with SRP programs.

In recent years, as the world’s most heavily polluted places come increasingly to be located in low- and middle-income countries (Blacksmith Institute 2013), SRP has directed substantial resources to study those environments. These efforts, undertaken in partnership with local universities and government agencies, seek to remediate problems in developing countries and also to learn lessons that can be applied in the United States. For example, the UC San Diego SRP represents SRP on the Good Neighbor Environmental Board (GNEB), an independent federal advisory committee that provides guidance to the President and the Congress on environmental challenges along the U.S.–Mexico border (U.S. EPA 2014). Another major SRP presence on the U.S.–Mexico border is the Dean Carter Binational Center for Environmental Health Sciences, a partnership between the University of Arizona, UC San Diego, and Texas A&M SRPs and 11 Mexican universities and research institutes (University of Arizona 2014). Its goal is to stimulate innovative cross-border research, community engagement, and education (U.S. EPA and Secretaría de Medio Ambiente y Recursos Naturales 2012). The Columbia University SRP has extensively studied the epidemiology and toxicology of arsenic in Bangladesh (Ahsan et al. 2006), the Harvard SRP has studied the consequences of lead exposure in Mexico (Gomaa et al. 2002; Téllez-Rojo et al. 2006), and the UC Berkeley SRP has studied the hematopoietic toxicity of benzene in China (Smith et al. 2005).

Finally, SRP goes beyond research to actually developing solutions to environmental health problems. Much work has gone into *a*) the development of novel technologies for detection of biologically active environmental chemicals, *b*) an understanding of the biological basis of environmental chemical toxicity, and *c*) the development of innovative remediation technologies to help clean up Superfund and other hazardous waste sites. The following are examples.

*New detection technologies*. Detection of aryl hydrocarbon agonists. Halogenated aromatic hydrocarbons (HAHs) are highly toxic and are found commonly in industrial waste. Michael Denison of the UC Davis SRP developed a new approach to track HAH and related dioxin-like chemical contaminants (Denison et al. 1998).

Using cultured cells, Denison manipulated the aryl hydrocarbon receptor system, the intricate biological mechanism that responds to HAHs, to create a highly specific, sensitive, and low-cost method for tracking dioxins and other HAHs (U.S. EPA and Secretaría de Medio Ambiente y Recursos Naturales 2012). This bioassay uses aryl hydrocarbon receptor signaling to link HAH contamination to the production of a fluorescent luciferase reporter. A further advantage of this bioassay is that it responds to HAH in a dose-dependent manner, and thus the relative brightness of the luciferase glow reflects the level of HAH-like activity. This work led to the development of the Chemical Activated LUciferase gene eXpression (CALUX®) system (Rogers and Denison 2000; Windal et al. 2005) now used by the U.S. EPA to evaluate HAH contamination in environmental media.

Detection of xenoestrogens. In August 2012, the BG1Luc assay, developed by Xenobiotic Detection Systems Inc., a system similar to the Denison system, and developed with support from an SRP SBIR grant, was accepted as part of EPA’s Tier 1 screening program for estrogenic chemicals (Gordon and Clark 2005). This assay provides a platform for testing the endocrine-disrupting activity of various compounds. It is based on hormone-stimulated transcriptional regulation and uses cultured cells that express estrogen receptors. When these receptors are stimulated by estrogenic compounds acting as agonists or antagonists, they modulate the expression of an estrogen promoter–regulated luciferase reporter gene.

Real-time analysis of multiple components in exhaust. A team of SRP researchers, led by Catherine Koshland at UC Berkeley, collaborated with scientists at Lawrence Berkeley National Laboratory to develop an entirely new technique for real-time, continuous monitoring of compounds commonly found in combustion effluents.

The technique uses excimer laser fragmentation fluorescence spectroscopy to detect and quantify multiple components in combustion exhausts (Avakian et al. 2002). High-energy photon beams fragment the contaminants that can then be optically detected by laser-induced fluorescence. The method can simultaneously detect multiple airborne elements and compounds down to very low levels (in the parts per billion range), including chlorinated hydrocarbons, barium, chromium, manganese, nickel, lead, and tellurium. The assay detects chemicals in real time and also *in situ* (Choi et al. 2005).

Before this invention, the standard practice for monitoring combustion by-products involved lengthy, labor-intensive sample extractions and preparations that could require several weeks.

*Understanding the biological basis of environmental chemical toxicity*. Arsenic. Arsenic is a natural contaminant of groundwater in nearly 70 countries around the world, including areas of the United States. It is also released to the environment by industrial processes such as mining, fossil fuel extraction, and hydraulic fracturing for natural gas. Arsenic is a proven human carcinogen. It also causes cardiovascular and other chronic diseases (States et al. 2009).

Bangladesh is very severely affected by arsenic in groundwater. An estimated 35–77 million people in Bangladesh depend on well water contaminated with arsenic. A multidisciplinary team from the Columbia University SRP established the Health Effects of Arsenic Longitudinal Study (HEALS) in Bangladesh to investigate and control health problems caused by chronic exposure to arsenic (Ahsan et al. 2006). HEALS is providing important data on arsenic toxicity across a wide range of exposure levels. A major finding is that chronic exposure to relatively low levels of arsenic is associated with an increase in overall mortality (Argos et al. 2010).

Another major finding is that prenatal exposure to arsenic in drinking water is negatively associated with brain development in children (Wasserman et al. 2011). This study, led by Joseph Graziano, found significant evidence for a negative dose–response relationship between arsenic exposure and childhood intelligence. The study found also that early-life exposure to arsenic affects motor function (Parvez et al. 2011). Children exposed to water arsenic concentrations > 10 μg/L performed more poorly on tests of intelligence and motor function than those exposed to < 10 μg/L. The present EPA drinking water standard for the United States is 10 μg/L (10 ppb) (U.S. EPA 2013a), and many regions around the world, including some areas of the United States, still have arsenic drinking water concentrations well above this standard.

The Columbia University team has leveraged additional resources to provide basic primary medical care to 20,000 people in rural Bangladesh. Before the team arrived, there were no physicians there. Now the clinic has its own pharmacy and has capacity to perform X-rays and electrocardiograms, and to provide dental care along with primary health care (States et al. 2009).

SRP research has demonstrated that arsenic exposure remains an important health issue in the United States. Margaret Karagas of the Dartmouth College SRP has led studies in the northeastern United States showing correlations between arsenic exposure in water and skin and bladder cancer (Karagas et al. 2002). Subsequent studies profiled the mechanisms for the arsenic-associated health effects including hypermethylation (Marsit et al. 2006a), DNA repair (Andrew et al. 2006), epigenetics (Marsit et al. 2006b), and genetic polymorphisms (Karagas et al. 2012).

Studies from the Dartmouth SRP were the first to report the presence of arsenic in rice and showed that rice consumption contributes to arsenic exposure among women in the United States (Gilbert-Diamond et al. 2011). These researchers found elevated levels of arsenic in brown rice syrup used in many processed foods, including many infant foods (Jackson et al. 2012).

The University of Arizona SRP has examined arsenic biotransformation and studied the impacts of arsenic metabolites on human health. Previously it was believed that methylation of arsenic was a detoxification process, but Vas Aposhian and colleagues showed that methylated metabolites of arsenic were, in fact, more toxic to cells than inorganic arsenic (Petrick et al. 2001). These researchers showed that the monomethylated metabolite of arsenic [monomethylarsonous acid (MMA^III^)] can be detected in urine (Marnell et al. 2003) and can be used to profile ethnic differences in the regulation of MMA^III^ production (Gomez-Rubio et al. 2012) as well as differences in body composition and lifestyle (Gomez-Rubio et al. 2011).

To investigate whether MMA^III^ is involved in the causation of bladder cancer, University of Arizona SRP researcher A. Jay Gandolfi exposed cultured human bladder cells to MMA^III^ at a concentration found in the urine of arsenic-exposed populations. He found that this exposure did indeed cause cellular changes consistent with malignant transformation (Eblin et al. 2008). Subsequent studies found that MMA^III^ imprinted cultured human bladder cells early in their continuous exposure (Wnek et al. 2010). These changes were shown to be irreversible and to be associated with changes in the epigenome (Jensen et al. 2009), cytokine signaling (Escudero-Lourdes et al. 2010), and global gene expression (Medeiros et al. 2012).

Benzene. To increase mechanistic understanding of benzene carcinogenicity, Stephen Rappaport, an exposure scientist, who was then at the University of North Carolina at Chapel Hill SRP, and Martyn Smith, a molecular toxicologist at the UC Berkeley SRP, developed and applied novel biomarkers of benzene exposure and at the same time investigated benzene-associated changes in DNA.

Rappaport and Smith have measured biomarkers in several hundred benzene-exposed workers and unexposed control subjects (Smith et al. 2005). They observed that low levels of exposure to benzene—levels < 1 ppm—cause a reduction in the number of circulating white blood cells and that benzene exposure is also toxic to hematologic progenitor cells, the unspecialized cells in the human bone marrow from which all blood cells develop (Zhang et al. 2012). This finding is very significant because the current occupational exposure standard for benzene in the United States and in many other countries is a time-weighted average exposure of 1 ppm. Rappaport et al. (2010) also found that benzene metabolite exposure biomarkers increase with increasing air concentrations, but in a nonlinear fashion, suggesting that cytochrome P450 metabolism of benzene is saturated at relatively low exposure levels.

The development by Rappaport et al. (2010) of a comprehensive set of biomarkers enabled these SRP researchers to systematically explore benzene–biomarker relationships over a wide range of occupational and environmental exposures. Their work was cited by EPA in justifying its decision to lower the benzene content of gasoline, pointing to supralinear (greater-than-proportional) production of benzene-related protein adducts at air concentrations < 1 ppm (U.S. EPA 2007).

Lead and metal mixtures. In 1994, a group of Harvard School of Public Health researchers led by Howard Hu initiated the Early Life Exposure in Mexico to Environmental Toxicants (ELEMENT) cohorts through an SRP grant (Harvard School of Public Health, Superfund Research Program 2014). The goal of this project was to map the adverse effects of lead exposure on early childhood development. Mexico City was chosen as the venue because lead exposure is very high there, reflecting use of leaded gasoline until 2000 and widespread use of lead-glazed pottery. Also, this location provided the opportunity to collaborate with a strong Mexican research team. Data from ELEMENT have confirmed the developmental neurotoxicity of lead at low levels, showed that measurement of bone lead content by X-ray fluorescence provides an accurate time-integrated biomarker of cumulative lead exposure, and demonstrated that lead from the maternal skeleton can be mobilized during pregnancy to increase maternal and fetal blood lead levels (Gomaa et al. 2002; Téllez-Rojo et al. 2006).

In 2006, Robert Wright, also then at Harvard, founded a second ELEMENT cohort in Mexico City focused on metal mixtures—arsenic, manganese, lead, and cadmium. This project is also quantifying exposures to social stress and poverty and exploring the hypothesis that combined exposures to metals and social stressors in early life have synergistic adverse effects on neurodevelopment. To date, the new ELEMENT study has detected a consistent pattern of adverse effects on development from both lead and social stress. It has also found that the disturbed salivary cortisol rhythms that follow high levels of stress are associated with adverse development (Braun et al. 2014).

Organophosphate neurotoxicity. Theodore Slotkin at the Duke University SRP is studying the effects on brain development and metabolism of exposures in early life to widely used organophosphate (OP) pesticides.

Data from Slotkin’s lab show that early-life OP exposure results in reduced numbers of neurons and in neurobehavioral deficits (Slotkin et al. 2006). To elucidate the underlying mechanisms, the Slotkin team evaluated gene expression in newborn rodents that had been exposed to low levels of the OP insecticides chlorpyrifos and diazinon during the first 4 days of life. They found that chlorpyrifos and diazinon each resulted in changes in expression of 20–25% of genes related to cell cycle and apoptosis pathways (Slotkin and Seidler 2012). Importantly, these effects were shown to be independent of cholinesterase inhibition, the primary mechanism for acute OP poisoning, and occurred at exposure levels below the threshold for cholinesterase inhibition.

Slotkin’s research team is also investigating the toxic effects of OP exposures on glucose and lipid metabolism. They found that early-life exposures of rodents to chlorpyrifos, diazinon, or parathion resulted in metabolic profiles resembling the initial stages of diabetes (Slotkin et al. 2005). More recently they observed that these exposures produce sex-selective changes in serum lipids, leading to increased body weight in adulthood (Slotkin 2011).

Further studies of OPs undertaken at the University of Washington SRP led by Clem Furlong and Lucio Costa have studied whether common single nucleotide polymorphisms (SNPs) in paraoxonase 1 (PON1), the gene that detoxifies chlorpyrifos (Shih et al. 1998), are related to increased susceptibility to cholinesterase inhibition. They found that the SNP at –108C results in doubling the concentration of PON1 in plasma, relative to the common form, –108T. Another SNP, Q192R, increases the catalytic efficiency of PON1 toward certain pesticides (Shih et al. 1998).

Furlong and Costa (2005) demonstrated that PON1 is slow to get “turned on” after birth. They have now begun to explore whether failure of PON1 to be expressed in early life might increase the vulnerability of young children to chlorpyrifos and other OP insecticides. Their studies found that children < 2 years old, especially those homozygous for PON1Q192, are potentially highly susceptible to chlorpyrifos toxicity (Furlong et al. 2005).

Organotins as environmental obesogens. A team of SRP investigators at Boston University, led by Jennifer Schlezinger, has used innovative techniques to assess plausible mechanisms through which organotins, used in anti-fouling paint on the hulls of ships, could stimulate formation of fatty bone tissue and degradation of structural bone (Bissonnette et al. 2010). Organotins are now ubiquitous in marine environments. Schlezinger demonstrated that extremely low doses of organotins adversely affect the development of B lymphocytes in the bone marrow through activation of the peroxisome proliferator–activated γ receptor (PPARγ) and the retinoid X receptor (RXR) (Yanik et al. 2011). Because this cell subset matures into blood lymphocytes responsible for generating protective antibodies, the results strongly suggested that organotins could act as immunosuppressants. Perhaps more important, the data also suggested that organotins have global effects on the bone microenvironment and on the development and function of other cell types that depend on the bone microenvironment for growth and differentiation signals.

With the understanding that PPAR controls fat (adipogenesis) and bone (osteogenesis) development, Schlezinger and her team reasoned that inappropriate activation of these receptors by organotins might skew differentiation of a bipotential mesenchymal stem cell toward the adipocyte lineage and away from the osteogenic lineage (Yanik et al. 2011). In fact, nanomolar concentrations of organotins appear to distort the differentiation of these stem cells so that formation of fat is favored over bone development and repair. Exposures to these chemicals thus resulted in premature aging of bone. Furthermore, the data suggest a plausible mechanism through which organotins could contribute to the accumulation of visceral fat—by inappropriately activating the PPAR in other organs.

PCB exposures and infant development. Harvard University SRP grantee Susan Korrick and her research team were among the first to find an association between *in utero* exposures to polychlorinated biphenyls (PCBs) and altered developmental achievement and growth in infancy and later childhood (Sagiv et al. 2008, 2012).

Their study was performed with a cohort of 900 mother–infant pairs in a community adjacent to a PCB-contaminated harbor and Superfund site in New Bedford, Massachusetts. It revealed that exposure to PCBs may be associated with adverse effects on fetal maturation and may therefore be a modifiable risk factor for premature birth. They also found that infant visual memory, which is predictive of cognitive performance in childhood, may also be impaired by *in utero* PCB exposure.

The researchers examined the children again when they were 8 years old. This follow-up investigation revealed that early-life exposures to PCBs were consistently associated with adverse behaviors, particularly behaviors associated with attention deficit/hyperactivity disorder (ADHD). They observed that sociodemographically disadvantaged children were especially susceptible (Sagiv et al. 2012).

These findings are notable because, although the cohort lives adjacent to a PCB-polluted harbor, PCB exposure levels in this population are similar to those found in other populations across the United States. This suggests that the developmental neurotoxicity of PCBs may occur at exposure levels found commonly.

PCB exposure and genetic variation in sentinel animals. An outstanding example of collaboration among SRP investigators is provided by a study undertaken by the Boston University SRP in partnership with Mark Hahn, a senior scientist at the Woods Hole Oceanographic Institution. Hahn has been using populations of fish in the New Bedford Harbor Superfund site to study the effects of environmental chemical exposure on genetic variation. He is trying to understand the genetic impacts of long-term, multi-generational exposure to high levels of dioxin-like chemicals on fish. Specifically, he is studying a population of estuarine fish, Atlantic killifish (*Fundulus heteroclitus*). Hahn demonstrated that New Bedford Harbor killifish are much less sensitive to dioxin-like compounds than are killifish from an uncontaminated reference site on Cape Cod and that this chemical resistance is heritable through several generations (Harbeitner et al. 2013; Oleksiak et al. 2011). He and his team have demonstrated that the gene pool of a highly exposed animal can be dramatically and possibly permanently changed following chronic exposure to PCBs.

Superfund chemicals, liver damage, and hepatocellular carcinoma. To examine the cellular and molecular mechanisms though which Superfund chemicals may cause liver damage and hepatocellular carcinoma, Michael Karin and his team in the UC San Diego SRP are studying environmental chemicals known to be mutagens. They found that initial injury to liver cells following exposures to these chemicals results in the generation of reactive oxygen species (ROS) (Sakurai et al. 2008). They further discovered that the IKK/NF-κB (I κB kinase/nuclear factor κB) inflammatory pathway within cells is critical to controlling levels of ROS (Karin and Greten 2005; Sakurai et al. 2006). In mice whose IKKβ in liver had been deleted, they demonstrated that ROS levels are increased. The consequence was high levels of apoptosis and cell death (He et al. 2010). Additionally, they found that these mice are at much higher risk of developing hepatocellular carcinoma when the mice are exposed to Superfund chemicals with mutagenic potential (Sakurai et al. 2006). These findings document that NF-κB is a critical mechanistic link between the onset of inflammation, liver toxicity, and long-term risk of cancer.

Further experiments conducted in the Karin laboratory have led to the discovery that in the presence of liver damage caused by mutagenic chemicals frequently found at Superfund sites, obesity can serve as a potent promoter of hepatocellular carcinoma (He et al. 2013; Park et al. 2010). This finding is of great significance for public health given the high prevalence of obesity in the United States.

Antimicrobials and endocrine disruption. Triclosan (TCS) and trichlocarban (TCC) are chlorinated phenols widely used as antimicrobials in personal care products such as soaps and disinfectants. TCC and TCS currently contaminate many waterways and are of growing concern to human and environmental health. Pharmacokinetic studies in humans have shown that TCC in commercial soap is rapidly absorbed through the skin and easily detectable in urine within 72 hr (Schebb et al. 2011).

Bruce Hammock in the UC Davis SRP has provided evidence in cell-based bioassay screens that TCC and TCS modify the estradiol- or testosterone-dependent activation of the estrogen receptor (ER) and the androgen receptor (AR) (Morisseau et al. 2009). In a collaboration with the UC San Diego SRC, Hammock and his team found that TCC treatment of mice activated both the constitutive androstane receptor and ERα, leading to downstream induction of target genes (Yueh et al. 2012). Hammock and his colleagues have recently shown that TCS alters type 1 ryanodine receptor (RyR1) activity and impairs physiological excitation–contraction coupling in both cardiac and skeletal muscle (Cherednichenko et al. 2012). TCC was also shown to be a potent inhibitor of soluble epoxide hydrolase, which has been linked to anti-inflammatory activity (Liu et al. 2011).

These findings provide important evidence that TCC and TCS can alter important hormonal signaling pathways in animals and humans. These data moved the Food and Drug Administration to recently reevaluate the benefit–risk ratio of using these agents in personal care products.

Nutritional modulation of Superfund pollutant toxicity. The University of Kentucky SRP studies mechanisms through which nutrition can modulate environmental insults. Dietary habits and particular foods are known to enhance and also to protect against the health impacts of exposures to environmental pollutants (Hennig et al. 2007).

Bernhard Hennig and his colleagues at Kentucky have shown that diets rich in proinflammatory fatty acids (e.g., omega-6 fatty acids) can exacerbate PCB-induced vascular endothelial activation and atherosclerosis (Hennig et al. 2002, 2005). In contrast, antioxidant and/or anti-inflammatory nutrients (e.g., omega-3 fatty acids and plant-derived polyphenols) protect against PCB toxicity (Majkova et al. 2011; Newsome et al. 2014).

Nutritional modulation of disease outcomes associated with exposure to Superfund pollutants is particularly important during critical developmental phases in early childhood. Much information is emerging related to epigenetic mechanisms involved in these processes (Hennig et al. 2012).

Phthalates and preterm births. One of the newest SRP Programs, at the University of Puerto Rico in partnership with SRP researchers at Northeastern University and the University of Michigan, is exploring possible links between environmental factors and preterm births. Leaders of this program, termed PROTECT—Puerto Rico Testsite for Exploring Contamination Threats—are José Cordero of the University of Puerto Rico, Akram Alshawabkeh of Northeastern University, and John Meeker and Rita Loch-Caruso of the University of Michigan. Preterm birth is a serious global public health problem that accounts for nearly 1 million infant deaths each year worldwide. Puerto Rico has the highest density of NPL sites per square mile and also the highest rate of preterm birth of any U.S. jurisdiction (Callaghan et al. 2006).

John Meeker from the University of Michigan showed that urinary phthalate metabolite concentrations were higher among preterm births in Puerto Rico than controls. He also found evidence for a positive dose–response relationship between preterm birth and exposures to some phthalates (Meeker et al. 2009). Meeker conducted a follow-on study using a nested case–control design in a much larger group of women that included 130 mothers who had delivered at < 37 weeks of completed gestation and 352 control mothers who delivered at ≥ 37 weeks. They found that women with the highest levels of phthalate exposure during pregnancy had up to five times the likelihood of preterm birth compared with women with the lowest exposure (Ferguson et al. 2014). These findings will be further explored in a longitudinal cohort of nearly 800 pregnancies in Puerto Rico.

Health consequences of disasters. Following the 11 September 2001 (9/11) terrorist attacks on New York City, SRP awarded emergency supplemental funding to the Mount Sinai School of Medicine SRP to study the health effects of the toxic environmental exposures that resulted from the destruction of the World Trade Center.

Three programs were launched: *a*) clinical and epidemiological studies of emergency response workers who served at Ground Zero, *b*) a prospective follow-up study of 182 pregnant women who were inside or near the World Trade Center at the time of the attacks, and *c*) a pediatric outreach program. These studies were launched in close coordination with the National Institute for Occupational Safety and Health and the New York City Department of Health and Mental Hygiene.

Two parallel occupational studies—one led by David Prezant that is following New York City Fire Department firefighters and paramedics, and the other by Philip Landrigan that is following the remainder of the 9/11 responder population—found that workers at Ground Zero developed new-onset cough, wheeze, and phlegm production in the initial weeks and months after the attacks (Landrigan et al. 2004; Weakley et al. 2011). These symptoms were most likely caused by inhalation of massive quantities of the highly alkaline dust (pH 10–11), which caused intense inflammation and subsequent scarring and distortion of airways. Ten-year follow-up assessment of this population found that despite a very low prevalence of tobacco smoking, 41% had persistently impaired pulmonary function, most commonly restrictive impairment. Pulmonary impairment and other abnormal findings were most frequent and severe in workers experiencing the heaviest exposure (Wisnivesky et al. 2011). Mental health symptoms were also common and they too showed an exposure–response gradient.

The major finding in the study of pregnant women led by Gertrud Berkowitz of Mount Sinai was that the rate of intrauterine growth restriction doubled in infants born to mothers who were inside or near the World Trade Center on 9/11, compared with unexposed controls in the New York City area (Berkowitz et al. 2003).

SRP also provided emergency supplemental funding to study health effects in the aftermath of Hurricane Katrina. Working through the NIEHS Portal, which combines advances in geographic information systems, data mining/integration, and visualization technologies, SRP investigators were able to provide decision makers in the Gulf Coast region with the data, information, and the tools they needed to *a*) monitor human and environmental health impacts of Hurricane Katrina; *b*) assess and reduce human exposures to contaminants; and *c*) develop science-based remediation, rebuilding, and repopulation strategies (Miranda 2007).

*Innovative remediation technologies*. Phytoremediation. Phytoremediation is the use of plants to remove, detoxify, or stabilize toxic chemicals in contaminated soils and groundwater. It represents a very attractive alternative to conventional remediation technologies, which are expensive and time consuming and can produce toxic by-products.

The late Milton Gordon and Lee Newman of the University of Washington SRP applied plant physiology, cell culture techniques, agronomy, microbiology, hydrogeology, and engineering to design a phytoremediation strategy for hazardous waste sites where groundwater is contaminated with organic solvents. They found that hybrid poplar trees can detoxify organic solvents such as trichloroethylene (TCE), perchloroethylene, and carbon tetrachloride that have become widespread industrial pollutants (Gordon et al. 1998).

SRP SBIR grantee Edenspace Systems Corp. is exploiting the ability of rabbit-foot grass *(Polypogon monospeliensis)* to remove mercury and sulfur from contaminated soils (Edenspace Systems Corporation 2014). The plant converts these elements to mercury sulfide, which has high stability and low solubility. Their work has focused primarily on wet ecosystems and has demonstrated high levels of mercury uptake by rabbit-foot grass to concentrations up to 110 times that of a control plant.

A multidisciplinary team from the University of Arizona SRP, led by environmental microbiologist Raina Maier and biogeochemist Jon Chorover, is using a phytoremediation approach to treat mining wastes. These wastes are prevalent in arid and semi-arid environments and can be highly contaminated with arsenic, lead, and other toxic metals. The team is using native plants that do not accumulate metals into shoot tissues to establish a vegetative cap and stabilize metals in the rooting zone—a process called phytostabilization (Mendez and Maier 2008). They have combined cutting-edge molecular ecology and geochemistry techniques to examine the plant root–microbe–tailings environment and the complex transitions that take place during revegetation. They are developing biogeochemical indicators to predict vegetation success. Major mining companies have partnered with this research team and will use these data to guide revegetation strategies.

To further boost the phytoremediation potential of plants, information is needed about the mechanisms and pathways that mediate the uptake, detoxification, and sequestration of toxic heavy metals (Mendoza-Cózatl et al. 2011; Song et al. 2010). Julian Schroeder’s laboratory at the UC San Diego SRP has identified several of the genes and proteins in plant vacuoles responsible for these actions. They function via ABC transporters and long-distance root-to-shoot transport mechanisms (Mendoza-Cózatl et al. 2011; Song et al. 2010).

Schroeder also discovered that a sodium metal transporter was key in protecting plants from salt stress, known to be a major factor in crop losses (Schroeder et al. 2013). Translating these findings to other investigators in Australia, the sodium transporters have been genetically engineered into wheat plants that are highly tolerant of ground salt. The outcome of this discovery, which was initiated through SRP efforts, has been an increase in wheat production of over 25%. By improving salt tolerance, important crops such as wheat can be grown on previously used farmland that has been claimed to be fallow due to salination. Such advances could eventually increase the world’s production of wheat and improve our planet’s food production.

Nanotechnology. Dibakar Bhattacharyya and other scientists at the University of Kentucky SRP developed a double-membrane remediation system that removes chlorinated organic contaminants from groundwater (Lewis et al. 2011). The device could offer an inexpensive way to provide clean drinking water in areas of the world where chemical contamination is prevalent. The system uses nanostructured materials to generate hydroxyl radicals that can remove the contaminants.

Bhattacharyya’s group also pioneered the synthesis of iron-based nanoparticles for toxic organic de-chlorination using “greener” approaches and responsive polymer membrane platform (Meeks et al. 2012).

Lindell Ormsbee and the University of Kentucky SRP Research Translation Core worked with Bhattacharyya to find ways to implement his technology at the Paducah Gaseous Diffusion Plant, Kentucky’s largest Superfund site, which contains significant contamination by TCE and PCB (Gui et al. 2013; Meyer et al. 2009).

Groundwater remediation. Mark Brusseau and his research group at the University of Arizona SRP have conducted an extensive, two decades–long, research program, comprising field tests, mathematical modeling studies, and laboratory investigations, focused on the Tucson International Airport Area Superfund site (NRC 2013). This program has investigated the transport and fate of chlorinated-solvent contaminants in groundwater, while addressing issues critical to the characterization and remediation of hazardous waste sites (NRC 2013).

These researchers have also developed and tested several innovative methods for characterization and remediation of subsurface contamination. For example, the first full-scale application of the partitioning tracer test method for detecting organic-liquid contaminants in the subsurface was tested at the site. In addition, some of the initial field applications of the integrated contaminant mass discharge test, the multi-solute diffusive tracer test, and the bioactive tracer test were conducted at the site (Brusseau et al. 2013).

Solar-powered electrochemical remediation. A team of researchers at the Northeastern University SRP, led by Akram Alshawabkeh, developed a solar-powered electrochemical technology to manipulate the chemistry of groundwater, enabling *in situ* treatment of contaminants (Alshawabkeh and Mao 2012). The technology uses solar panels to apply low-level direct electric currents through electrodes in wells, manipulating the groundwater chemistry through electrolysis to create conditions favorable for either reduction or oxidation of contaminants. The process is versatile and can treat a mixture of chemicals including TCE, dichromate, selenite, nitrate, and phosphate.

This technology offers several advantages: *a*) It is driven by a renewable energy source, *b*) it is environmentally friendly because it does not require the addition of chemicals into groundwater, and *c*) rates of redox (oxidation-reduction) reactions can be controlled by adjusting electric current intensity.

Training programs. Training programs that educate the next generation of leaders in environmental health science have been an essential component of SRP since its inception. Each academically based SRP is required to contain a training program that supports graduate- and postdoctoral-level cross-disciplinary training in fields related to environmental health and environmental science and engineering. SRP regards these training programs as a vital for the mentorship, education, and training of environmental health and science professionals.

Over the past 25 years, SRP has trained a total of 1,504 trainees in 32 institutions. Of these, 689 are currently in training, and 815 have completed training. The scientists and engineers who have finished their training have pursued a range of careers—267 in academia, 45 in government, 143 in industry, and 49 in other areas.

SRP has established two awards for outstanding trainees. The Karen Wetterhahn Memorial Award, established in 1998, recognizes an outstanding young research scientist conducting research relevant to SRP who demonstrates the qualities of scientific excellence exhibited by Karen E. Wetterhahn (1948–1997), professor of chemistry and founder of the Dartmouth College SRP. Wetterhahn was an internationally recognized chemist who studied mechanisms of metal toxicity. Tragically, she died in 1997 as the result of a laboratory accident in which a few drops of highly toxic liquid dimethylmercury penetrated her protective latex glove and caused rapidly progressive, fatal neurological impairment. Wetterhahn was deeply concerned with bringing young women into science. She was co-founder of the Women in Science Project at Dartmouth, a highly successful program where first-year women engage in learning experiences designed to further their interest in science, mathematics, or engineering.

The KC Donnelly Externship Award, named in honor of Kirby Cornwall (KC) Donnelly (1951–2009) of Texas A&M University, provides opportunities for current SRP-funded graduate students and postdoctoral researchers to compete for translational externships outside of their home universities. Donnelly was professor and head of the Department of Environmental and Occupational Health, and Associate Director of the Texas A&M SRP. As leader of the South Texas Environmental Education and Research (STEER) program, he helped bridge the gap between public health and medicine for > 500 students (University of Texas Health Science Center at San Antonio 2015). In his environmental research on the U.S.–Mexico border, Donnelly stressed the importance of learning coupled with community-based research as he worked to improve the lives of poor and disenfranchised border populations exposed to hazardous waste.

## Discussion

The problem of hazardous waste, though much better controlled in the United States today than in the past, has not gone away. New chemicals and new technologies are invented every year (Landrigan and Goldman 2011). New forms of waste are created. Hazardous chemical and nuclear wastes continue to pose major threats to the environment and to public health. Laws and regulations on chemical safety are relatively weak (Landrigan and Goldman 2011).

Hazardous waste and chemical pollution have also become global issues. Production of synthetic chemicals and the generation of hazardous waste were initially concentrated in high-income countries, reflecting the origins of the chemical manufacturing industry in Western Europe in the late 19th and early 20th centuries and its spread in the 20th century to North America, Japan, and Australia. Today, however, the chemical manufacturing industry (Goldstein et al. 2013) is increasingly globalized, and the manufacture and use of chemicals are shifting increasingly to low- and middle-income countries, where labor costs are low and environmental protections often few. Also, exports to low-income countries of hazardous materials such as asbestos (World Health Organization 2014), banned pesticides (Smith et al. 2008), and e-waste (Robinson 2009) are accelerating. As a result, the threat of hazardous waste is greatest today in low- and middle-income countries (Caravanos et al. 2011; Grant et al. 2013), and in those countries, hazardous waste has become an important, although insufficiently recognized, contributor to the burden of disease (Chatham-Stephens et al. 2013).

Tragic episodes of human exposure to toxic chemicals have resulted from the migration of the chemical manufacturing industry and toxic chemicals to developing countries. These include acute episodes such as the Bhopal disaster in India, where thousands of people of all ages died or were severely injured after exposure to methyl isocyanate released by an explosion in a pesticide manufacturing facility (Dhara et al. 2002), as well as chronic, slowly unfolding tragedies such as the current exposure of > 1 million persons in China, South and Southeast Asia, and sub-Saharan Africa to chrysotile asbestos (Frank and Joshi 2014).

In industrially developed as well as developing countries, the populations at greatest risk of exposure to toxic chemicals are those who live or work near polluting industries and hazardous waste sites (U.S. EPA 1996). In the United States, these populations include the 11 million people who live within 1 mile of a federal Superfund site, the many others who live in proximity to state Superfund sites, and the many thousands of hazardous waste workers and emergency responders who work at these sites or respond to fires and other emergencies at them.

Children living in communities near hazardous waste sites are at especially high risk because their unique patterns of exposure and heightened biological vulnerability increase the likelihood that they will be exposed to toxic chemicals and suffer adverse health effects (Landrigan et al. 1999; NRC 1993).

Poor and minority populations are at high risk of exposure because hazardous waste sites are disproportionately located in neighborhoods of lower socioeconomic status and predominantly minority demographics—a circumstance termed “environmental injustice” (Faber et al. 2002; United Church of Christ Commission for Racial Justice 1987).

The SRP commitment to community engagement has allowed for the creation of partnerships with community groups most seriously affected by contamination. SRP has supported the development and use of innovative tools for communication and engagement (Pezzoli et al. 2007). One such tool, created by the Boston University SRP, is a guide for community groups (Scammell and Howard 2015). This guide helps communities decide whether a health study is an appropriate strategy for addressing their concerns. It is freely available and includes contributions by residents living near contaminated sites, community and agency partners, and SRP investigators. A second example is the freely available, transferrable training modules developed by the University of Arizona SRP in collaboration with promoters (Hispanic community health workers) (University of Arizona, Superfund Research Program 2015). These modules are based on a “train the trainer” model and provide information to communities on how to understand and reduce exposures to toxicants.

## Conclusion

The NIEHS SRP program was created 25 years ago in response to a crisis—the discovery that hazardous waste was a major and previously unrecognized threat to human health and the environment. That threat remains, and it is now spreading from the United States, Japan, Australia, and Western Europe to low- and middle-income countries, where it is an increasingly important, but insufficiently recognized, risk factor for disease.

In the years since its founding, SRP has grown modestly in size and greatly in scientific credibility and real-world impact. SRP is a small program, but it has had an outsized scientific influence. Its record is one of significant scientific achievements accomplished with extremely modest investment combined with clever leveraging of resources from many sources. The program has been extraordinarily effective in facilitating research innovation, in catalyzing interdisciplinary and even transdisciplinary research, and in translating research to stakeholders, including regulators, industry, and affected communities.

Many factors have contributed to the scientific stature of SRP research, but four stand out.

The first is the fact that SRP provides targeted funds to investigate the health effects of hazardous waste—an area of scientific investigation that otherwise would likely be neglected.

The second is the program’s base in a peer-reviewed, competitively awarded grant program. The NIH peer review system, which has been central to the rise of American science in the past half century, is critical to the success of SRP. It encourages and supports excellent scientists and draws some of the nation’s best minds to focus on complex problems relevant to hazardous waste.

The third critical factor is the program’s unique transdisciplinary structure that brings together biomedical researchers and engineers, ecologists, earth scientists, social scientists, and evolutionary biologists. Because of this program, these seemingly disparate fields have now become connected in universities across the United States. The extraordinary cross-pollination achieved through this unique model has been essential to the program’s success, producing innovations that no single scientific discipline could have achieved on its own.

Skilled and experienced leadership at NIEHS has been the fourth factor critical to the success of the SRP. Beginning with David P. Rall, NIEHS Director at the time of the establishment of SRP, and extending through all the successive Directors up to and including the present NIEHS Director, Linda Birnbaum, the SRP has enjoyed high-level support from top management of NIEHS. Just as important has been the long time leadership of SRP’s founding Program Director, William Suk (Wilson 2014).

If past is prologue, SRP’s first 25 years have provided a firm foundation in the face of an uncertain future for United States science. Whether we can continue to develop more and better science to solve one of the world’s most difficult problems remains to be seen. But the potential is there, and a quarter-century of success has shown that the resolution of pressing environmental issues through outstanding science is highly feasible, even in these resource-scarce times, with the most modest of investments.
